# Supplementary material for: Content-rich biological network constructed by mining PubMed abstracts
Source: BMC Bioinformatics. 2004 Oct 8;5:147. doi: 10.1186/1471-2105-5-147 (PMC528731; doi:10.1186/1471-2105-5-147)
Supplement: Additional File 2 — The original results of the above study (non-essential files are deleted to keep the file size under the limit set by BMC bioinformatics). [file 1471-2105-5-147-S2.bz2 › chilibotAdditionalFile2/dip05/680ID7658473E11090/html/CTXA_CTXB.html]

 


 **CTXA** and **CTXB** 
  
Found 12 abstracts in PubMed, retrieved 05.  
 

 What does Google say? 
 PDF only 
| .edu only 

---

**Interactive relationship** (e.g. stimulation, inhibition, etc)

**Non-interactive relationship** (e.g. studied together, co-existance, homology, etc.)

- sequence data were also obtained for the virulence associated genes tcpA,  **ctxA** , and  **ctxB** .  Ref: 12734277 J Clin Microbiol, 2003
- Among these were three V. cholerae strains that contained CTX prophage sequences but not the  **ctxA**  and  **ctxB**  genes.  Ref: 10986258 J Bacteriol, 2000
- The genes encoding cholera toxin  **ctxA**  and  **ctxB**  are encoded in the genome of CTXphi, a filamentous phage that infects Vibrio cholerae.  Ref: 10986258 J Bacteriol, 2000
- PCR analysis showed that 71% of O1 clinical isolates had a complete set of CTX element target genes  **ctxA** ,  **ctxB** , zot and ace and 68% a complete set of the VPI genes studied orf1, aldA, tagA, tcpA, toxT and int genes.  Ref: 12393195 FEMS Microbiol Lett, 2002
- Comparison of cholera toxin CT production directed by different gene constructs and S1 nuclease mapping revealed the presence of a  **ctxB**  specific promoter within the  **ctxA**  coding sequence.  Ref: 9119505 Infect Immun, 1997
- PCR amplification with primers specific to the  **ctxA** ,  **ctxB** , tcpA, tagA, int, aldA, toxT, LJ, RJ and toxR genes revealed that six of the seven rough isolates were positive for these genes.  Ref: 11232774 J Med Microbiol, 2001
- The results also showed that 72.4% of environmental O1 isolates possessed  **ctxA** ,  **ctxB** , zot and ace genes while environmental non O1 strains rarely possessed virulence genes.  Ref: 12393195 FEMS Microbiol Lett, 2002
